# Exposure to 1800 MHz LTE electromagnetic fields under proinflammatory conditions decreases the response strength and increases the acoustic threshold of auditory cortical neurons

**DOI:** 10.1038/s41598-022-07923-9

**Published:** 2022-03-08

**Authors:** Samira Souffi, Julie Lameth, Quentin Gaucher, Délia Arnaud-Cormos, Philippe Lévêque, Jean-Marc Edeline, Michel Mallat

**Affiliations:** 1 Paris Saclay Institute of Neuroscience, Neuro-PSI, UMR 9197 CNRS, Université Paris-Sud, 91405 Orsay Cedex, France; 2 CNRS, XLIM, UMR 7252, Univ. Limoges, 123 avenue Albert Thomas, 87000 Limoges, France; 3 Institut Universitaire de France (IUF), 1 rue Descartes, 75005 Paris, France; 4 Institut du Cerveau - Paris Brain Institute, ICM, Inserm, CNRS, Sorbonne Université, APHP, Hopital Pitie Salpetrières, 75013 Paris, France

**Keywords:** Auditory system, Glial biology, Neuroimmunology, Sensory processing, Neuroscience

## Abstract

Increased needs for mobile phone communications have raised successive generations (G) of wireless technologies, which could differentially affect biological systems. To test this, we exposed rats to single head-only exposure of a 4G long-term evolution (LTE)-1800 MHz electromagnetic field (EMF) for 2 h. We then assessed the impact on microglial space coverage and electrophysiological neuronal activity in the primary auditory cortex (ACx), under acute neuroinflammation induced by lipopolysaccharide. The mean specific absorption rate in the ACx was 0.5 W/kg. Multiunit recording revealed that LTE-EMF triggered reduction in the response strength to pure tones and to natural vocalizations, together with an increase in acoustic threshold in the low and medium frequencies. Iba1 immunohistochemistry showed no change in the area covered by microglia cell bodies and processes. In healthy rats, the same LTE-exposure induced no change in response strength and acoustic threshold. Our data indicate that acute neuroinflammation sensitizes neuronal responses to LTE-EMF, which leads to an altered processing of acoustic stimuli in the ACx.

## Introduction

The electromagnetic environment of human populations has substantially evolved over the last three decades owing to the continuous expansion of wireless communications. Currently, more than two-thirds of the human population is considered as mobile phone (MP) users. The massive spreading of this technology has nourished concerns and debates about possible hazardous effect of pulsed electromagnetic fields (EMF) in the radiofrequency (RF) range, which are emitted by MP or base stations and encode the communications. This public health issue has stimulated a wealth of experimental research dedicated to the effects of RF absorption in biological tissues^1^. Parts of these investigations have looked for alterations of neuronal network activities and cognitive processes, considering the proximity of the brain to the source of RF under common use of MP. Many of the reported studies pertains to the influence of pulse-modulated signals used in the second generation (2G) global system for mobile communication (GSM), or in the wideband code-division multiple access (WCDMA)/3rd Generation Universal Mobile Telecommunication system (WCDMA/3G UMTS)^2–5^. Much less is known about the effects of RF signals used in the fourth generation (4G) mobile services, which rely on a fully digital Internet protocol-based technology called long term evolution (LTE) technology. The LTE mobile phone service was launched in 2011 and the worldwide number of LTE subscriptions was estimated to reach 6.6 billion in January 2022 (Global mobile suppliers associations: //gsacom.com). In contrast with the GSM (2G) and WCDMA (3G) systems that are based on single-carrier modulation schemes, LTE uses orthogonal frequency division multiplexing (OFDM) as the basic signal format^6^. Worldwide, LTE mobile services utilize a range of different frequency bands comprised between 450 and 3700 MHz including the 900 and 1800 MHz bands also used in the GSM.

The capacity of RF exposure to impact on biological processes strongly depends on the specific absorption rate (SAR) expressed in W/kg, that measures the energy absorbed in the biological tissues. The effect of an acute 30 min head exposure to a 2.573 GHz LTE signal on the global neuronal networks activity was recently explored in healthy human volunteers. Using resting state functional resonance magnetic imaging, it was observed that the LTE exposure could induce alterations in spontaneous slow frequency fluctuations and in intraregional or interregional connectivities, whereas spatial peak SAR levels averaged over 10 g of tissue were estimated to vary from 0.42 to 1.52 W/kg, according to subjects^7–9^. Under similar exposure conditions (30 min duration, peak SAR level estimated at 1.34 W/kg using a representative human head model) electroencephalographic analyses indicated reduced spectral powers and interhemispheric coherences in the alpha and beta bands^10^. However, two other studies based on EEG analyses found that 20 or 30 min LTE head exposures with maximum local SAR levels set around 2 W/kg had either no detectable effect^11^ or resulted in reduced spectral power in the alpha band, with no alterations in cognitive functions assessed with the Stroop test^12^. Significant discrepancies were also noted in the outcome of EEG or cognition studies dedicated to the effect of GSM or UMTS EMF exposures. They were thought to originate from variation in methodological designs and experimental parameters including signal types and modulations, exposure intensity and duration, or from heterogeneity among the human subjects with respect to age, anatomy or gender^2,5,13^.

So far, animal studies have been seldom used to determine how exposure to LTE signals could affect brain functions. It was recently reported that whole body exposures of developing mice from late embryonic stages to weaning (30 min/day, 5 days/week, whole body average SAR of 0.5 or 1 W/kg) can cause altered locomotion and appetitive behaviors in adult life^14^. Repeated whole body exposures of adult rats (2 h a day for 6 weeks) were found to trigger oxidative stress and reduced the amplitude visual evoked potential obtained from the optic nerve, while the maximum SAR was estimated to be as low as 10 mW/kg^15^.

Besides analyses at multiple scales including cellular and molecular levels, rodent models can be used to investigate the effects of RF exposures during diseases, as illustrated by previous studies dedicated to the impact of GSM or WCDMA/3G UMTS EMF in the context of acute neuroinflammation, seizures, neurodegenerative diseases or glioma^16–20^.

Lipopolysaccharide (LPS)-injected rodent is a classical preclinical model of an acute neuroinflammatory reaction associated to benign infectious diseases, which are caused by virus or bacteria and affect most of the human population each year. This inflammatory state is responsible for a reversible sickness and depressive behavior syndrome marked by fever, hypophagia and reduced social interactions^21^. Residents CNS phagocytes such as microglia are key effector cells in this neuroinflammatory reaction. Rodent treatment with LPS triggers activation of microglia marked by remodeling of their shape and cell processes as well as deep changes in their transcriptome profile including upregulation of genes encoding proinflammatory cytokines or enzymes, which impact on the activity of neuronal networks^22–24^.

Investigating the effects of a single 2 h head exposure to GSM-1800 MHz EMF in LPS-treated rats, we found that GSM signals trigger cell responses in the cerebral cortex affecting expression of genes, phosphorylation of glutamate receptors, neuronal evoked discharges and microglial cell morphology in the cerebral cortex. These effects were not detected in healthy rats submitted to a same GSM exposure indicating that the LPS-triggered neuroinflammatory state sensitize CNS cell responses to GSM signals^25–28^. Focusing on the auditory cortex (ACx) of LPS-treated rats in which the local SAR averaged 1.55 W/kg, we observed that GSM-exposure resulted in an increased length or branching of microglia processes together with reduced neuronal responses evoked by pure tones and natural stimuli^28^.

In the current study, we aimed at examining whether a head-only exposure to LTE-1800 MHz signals could also alter microglial cell morphologies and neuronal activity in the ACx, reducing the power of the exposure by two-thirds. We show here that in the ACx of LPS-treated rats where the SAR value was 0.5 W/kg, LTE signals had no effect on microglial cell processes but, still, triggered significant reduction in sound-evoked cortical activity.

## Results

### LTE exposure does not affect microglial cell morphology in the auditory cortex

Given previous demonstration that exposure to GSM-1800 MHz alters microglial cell morphology under proinflammatory conditions, we investigated this effect after exposure to LTE signals.

**Figure 1:**
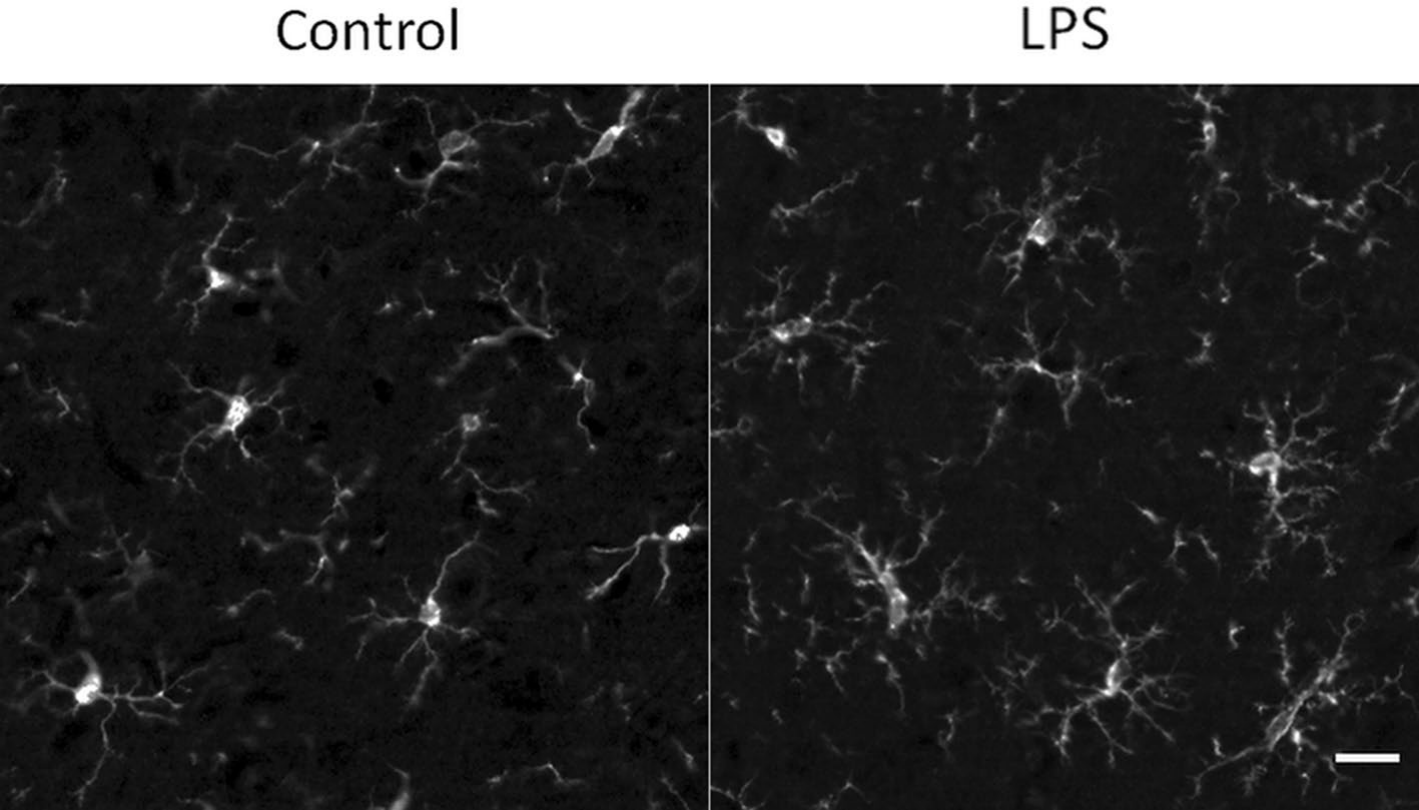
Effect of LPS ip injection on the morphology of cortical microglia. Representative views of microglia in coronal sections of the cerebral cortex (dorso medial area), 24 h after i.p. injections with LPS or vehicle (Control). The cells were stained with anti-Iba1 antibody as described previously^26^. The LPS proinflammatory treatment resulted in changes in microglia cell morphologies including proximal thickening and increased short secondary branching of the cell processes leading to a “bushy like” appearance. Scale bar: 20 µm.

**Figure 2:**
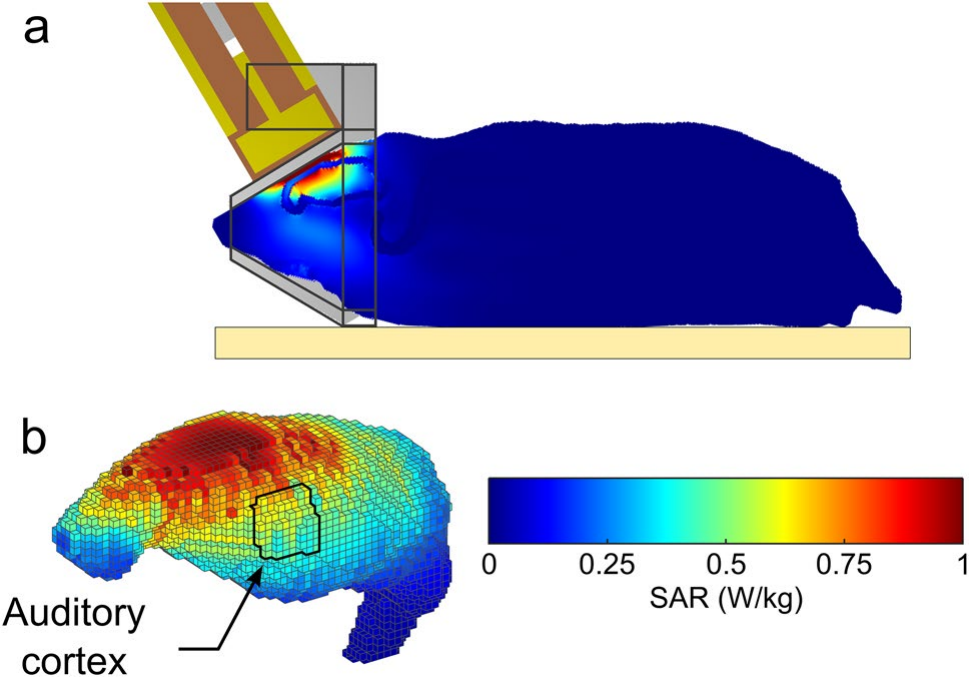
Dosimetric analysis of Specific Absorption Rates (SARs) in the rat brain during exposure to 1800 MHz LTE. The heterogeneous model of phantom rat and loop antenna previously described^62^ was used to evaluate the local SAR in the brain with a 0.5 mm^3^ cubic mesh. (**a**) Global view of the rat phantom in the exposure setup with the loop antenna above its head and the metal-free heat pad (yellow) under the body. (**b**) Distribution of SAR values in the adult brain at 0.5 mm^3^ spatial resolution. The areas delimited by black contours in sagittal sections correspond to the primary auditory cortex in which microglia and neuronal activity were analyzed. The color-coded scale of SAR values applies to all numerical simulations shown in the figure.

**Figure 3:**
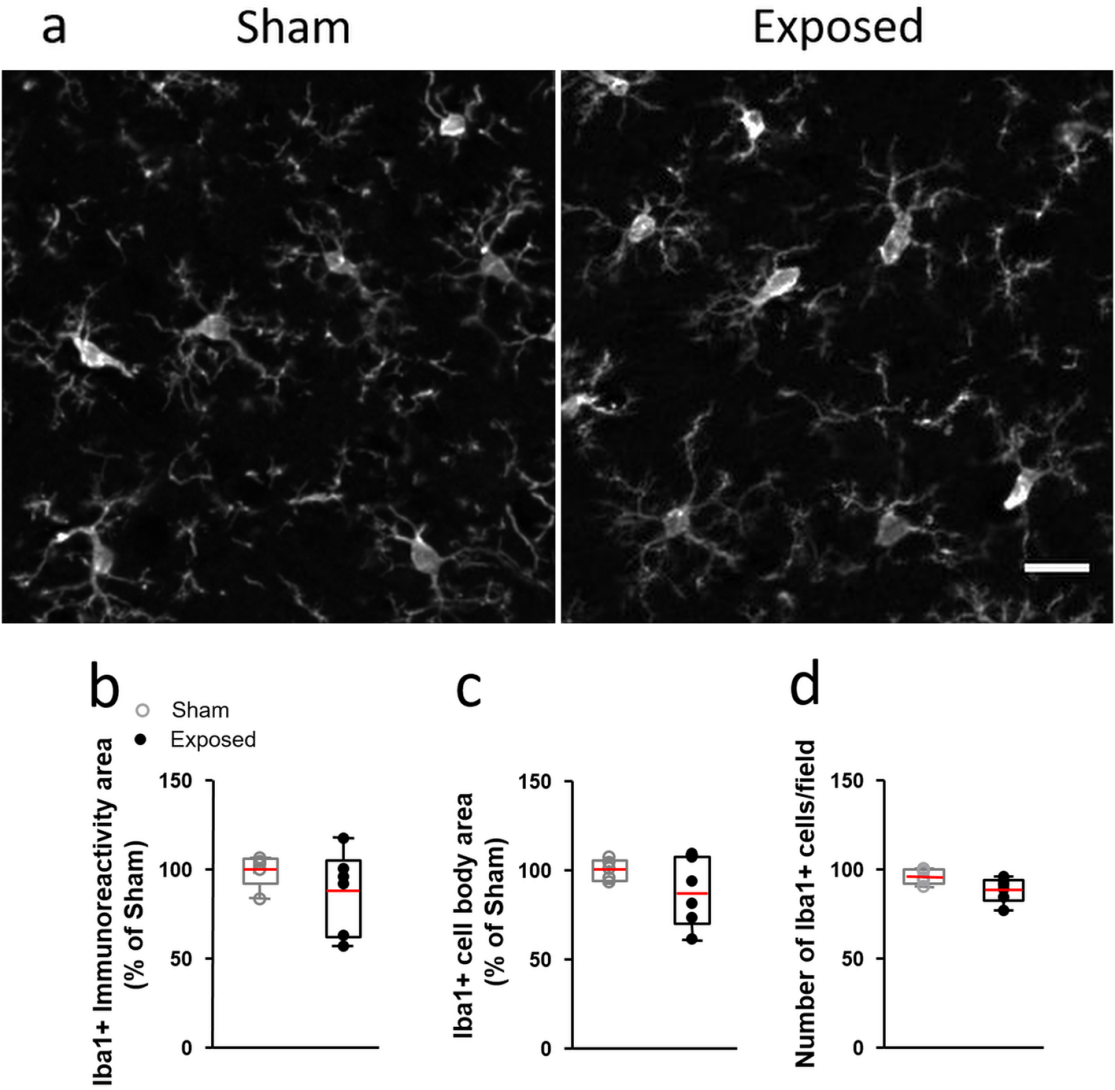
Microglia in the auditory cortex of LPS-injected rats after LTE or Sham exposure. (**a**) Representative stacked views of microglia stained with anti–Iba1 antibody in coronal sections of the auditory cortex from LPS–injected rats that were perfused 3 to 4 h after Sham or LTE exposure (exposed). Scale bar: 20 µm. (**b**–**d**) Morphometric assessment of microglia 3 to 4 h after sham (open dots) or LTE–exposure (exposed, dark dots). (**b**,**c**) Spatial coverage of the microglial marker Iba1 (**b**) and area of Iba1-positive cell bodies (**c**). Data represents the area of anti-Iba1 staining normalized to mean values from Sham-exposed animals. (**d**) Counts of anti-Iba1-stained microglial cell bodies. Differences between Sham (n = 5) and LTE (n = 6) animals were not significant (p > 0.05, unpaired t- test). The top and bottom of the box, and the upper and lower lines indicate 25th–75th percentiles and 5th–95th percentile respectively. The mean value is marked in red in the box.

Adults rats were injected with LPS 24 h before a head-only Sham-exposure or an exposure to LTE-1800 MHz. At the time of exposure, the LPS-triggered neuroinflammatory reaction was established in the cerebral cortex as indicated by upregulation of proinflammatory genes^26^ and by the changes in the morphology of cortical microglia (Fig. 1). The power of the LTE head- exposure was set in order to get a mean SAR level of 0.5 W/kg in the ACx (Fig. 2). To determine whether LPS-activated microglia reacted to LTE EMF, we analyzed cortical sections stained with anti-Iba1 that selectively labels these cells^29^. As illustrated in Fig. 3a microglial cells looked very similar in sections of ACx fixed 3 to 4 h after sham or LTE exposure, showing the “bushy like” cell morphology elicited by the LPS proinflammatory treatment (Fig. 1). Consistent with an absence of morphological response, quantitative image analyses showed no significant differences in the total area of Iba1 immunoreactivity (unpaired t-test, p = 0.308) or in the area (p = 0.196) and the density (p = 0.061) of Iba 1-stained cell bodies, when comparing LTE-exposed rats with Sham-exposed animals (Fig. 3b–d).

### LTE exposure decreases neuronal responses in LPS-treated animals

Table 1 summarizes the number of animals and multi-unit recordings obtained in the primary auditory cortex for the four groups of rats (Sham, Exposed, Sham-LPS, Exposed-LPS). In the following results, we have included all recordings exhibiting significant spectro-temporal receptive fields (STRFs), that is tone-evoked responses at least six standard deviations above spontaneous firing rate (see Table 1). Applying this criterion, we selected 266 recordings for the Sham group, 273 recordings for the Exposed group, 299 recordings for the Sham-LPS group and 295 recordings for the Exposed-LPS group.

**Table 1:** Summary of the number of animals and selected cortical recordings in LTE-exposure and LPS-treatment conditions.

	Sham	Exposed	Sham + LPS	Exposed + LPS
Number of animals	5	5	5	5
Number of multiunit-recordings	512	496	400	416
Number of multiunit-recordings with a STRF	266	273	299	295
Number of array positions	32	31	25	26

In the following paragraphs, we will first describe the parameters extracted from the spectro-temporal receptive fields (i.e., the responses to pure tones) and from the responses to heterospecific vocalizations. Then, we will describe the quantifications of frequency response areas obtained for each group. To take into account the existence of “nested data”^30^ in our experimental design, all the statistical analyses were performed based on the number of positions of the electrode-array (last line in Table 1), but all the effects described below were also significant based upon the total number of multi-unit recordings collected in each group (third line in Table 1).

**Figure 4:**
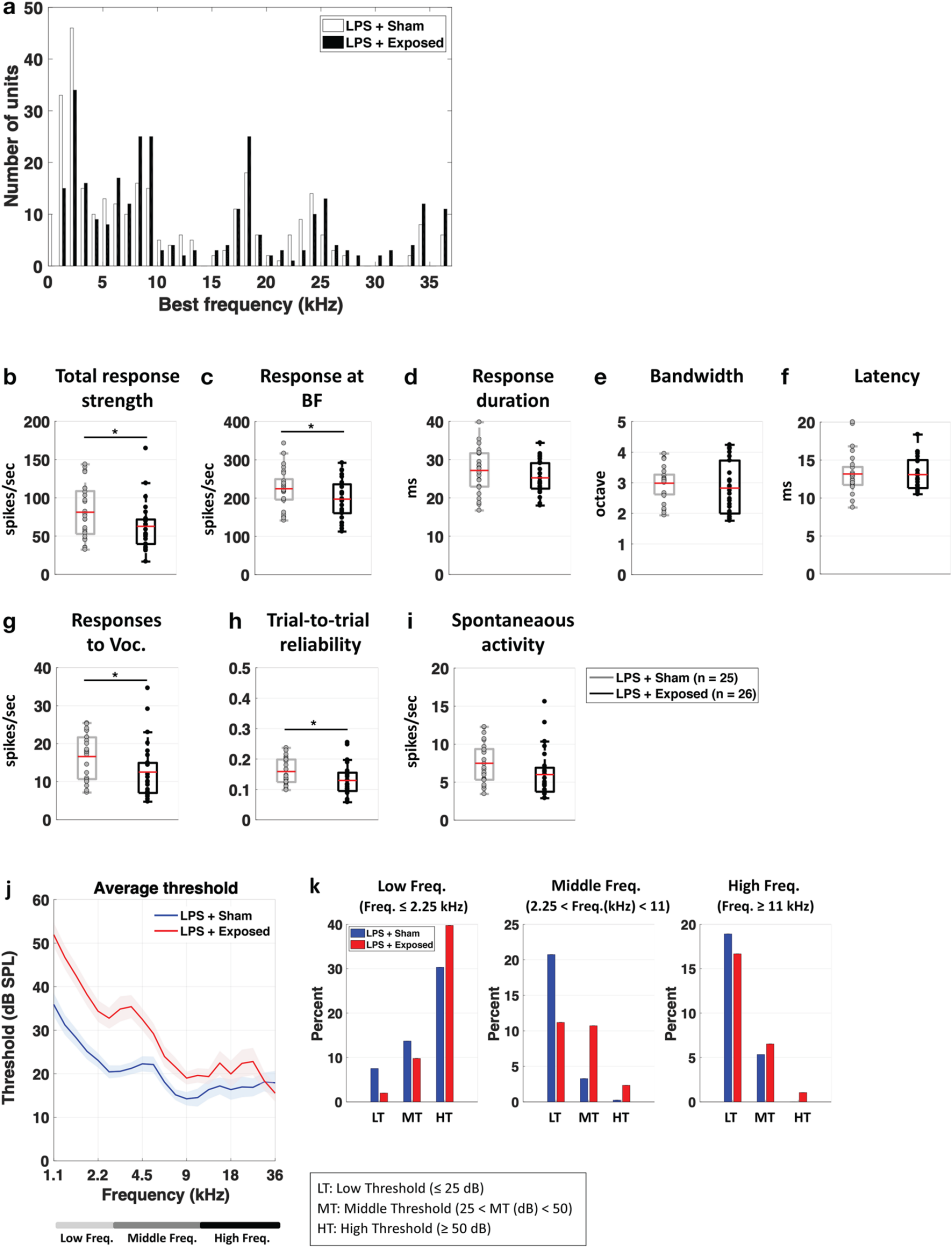
Effects of LTE exposure on parameters quantified from cortical responses in the LPS-treated animals. (**a**) BF distributions of the cortical neurons for the LPS-treated animals exposed to LTE (in black) and Sham-exposed to LTE (in white). The two distributions did not differ. (**b**–**f**) Effects of LTE exposure on the parameters quantifying the spectro-temporal receptive fields (STRF). The response strength was significantly decreased (* p < 0.05, unpaired t test) both on the entire STRF (total response strength) and at the best frequency (**b**,**c**). The response duration, the response bandwidth and the bandwidth were unchanged (**d**–**f**). Both the strength and the temporal reliability of the responses to vocalizations were decreased (**g**,**h**). Spontaneous activity was not significantly decreased (**i**). (* p < 0.05, unpaired t-tests). (**j**,**k**) Effects of LTE exposure on the cortical thresholds. The average thresholds were significantly higher in LTE-exposed rats compared to Sham-exposed rats. This effect was more pronounced in the low and middle frequencies.

Figure 4a presents the distributions of best frequencies (BF, eliciting the largest responses at 75 dB SPL) for the cortical neurons obtained in the Sham and Exposed animals treated with LPS. The frequency range of the BFs extended from 1 to 36 kHz in both groups. Statistical analysis showed that these distributions were similar (Chi-Square, p = 0.278) indicating that comparisons between the two groups can be performed without sampling bias.

Figure 4b–f shows the distributions (means are represented by red lines) of the parameters derived from the STRFs for these animals. The effects induced by LTE exposure on the LPS-treated animals seem to point out a decrease in neuronal excitability. First, the total response strength and the response at the BF were significantly decreased compared to the Sham-LPS animals (Fig. 4b,c unpaired t tests, p = 0.0017; and p = 0.0445). Similarly, the responses to communication sounds were decreased both in terms of response strength and of trial-to-trial reliability (Fig. 4g,h; unpaired t test, p = 0.043). Spontaneous activity was decreased but this effect was not significant (Fig. 4i; p = 0.0745). The response duration, the tuning bandwidth and the response latencies were not affected by LTE exposure in the LPS treated animals (Fig. 4d–f) suggesting that the frequency selectivity and the precision of onset responses were not impacted by LTE exposure in LPS-treated animals.

We next assessed whether the pure-tone cortical thresholds were altered by the LTE-exposure. Based on the Frequency Response Areas (FRAs) obtained from each recordings, we determined the auditory threshold at each frequency and averaged these thresholds in the two groups of animals. Figure 4j shows the mean (± sem) thresholds for the LPS treated rats from 1.1 to 36 kHz. Comparing the auditory thresholds of the Sham and Exposed groups revealed large increases in threshold in the Exposed animals compared to the Sham animals (Fig. 4j), an effect that was more prominent in the low and middle frequencies. More precisely, in low frequencies (< 2.25 kHz), the proportions of A1 neurons with high thresholds increased and the proportion of low and mid-thresholds neurons decreased (Chi-Square = 43.85; p < 0.0001; Fig. 4k, left panel). The same effect was also present in the middle frequencies (2.25 < Freq(kHz) < 11): there was higher proportion of cortical recordings with mid thresholds and a smaller proportion of neurons with low thresholds compared to the non-exposed group (Chi-Square = 71.17; p < 0.001; Fig. 4k, middle panel). There was also a significant difference in terms of threshold for the high frequency neurons (≥ 11 kHz, p = 0.0059); the proportion of neurons with low threshold decreased and the proportion middle and high threshold increased (Chi-Square = 10.853; p = 0.04 Fig. 4k, right panel).

### LTE exposure alone did not change the response strength in healthy animals

**Figure 5:**
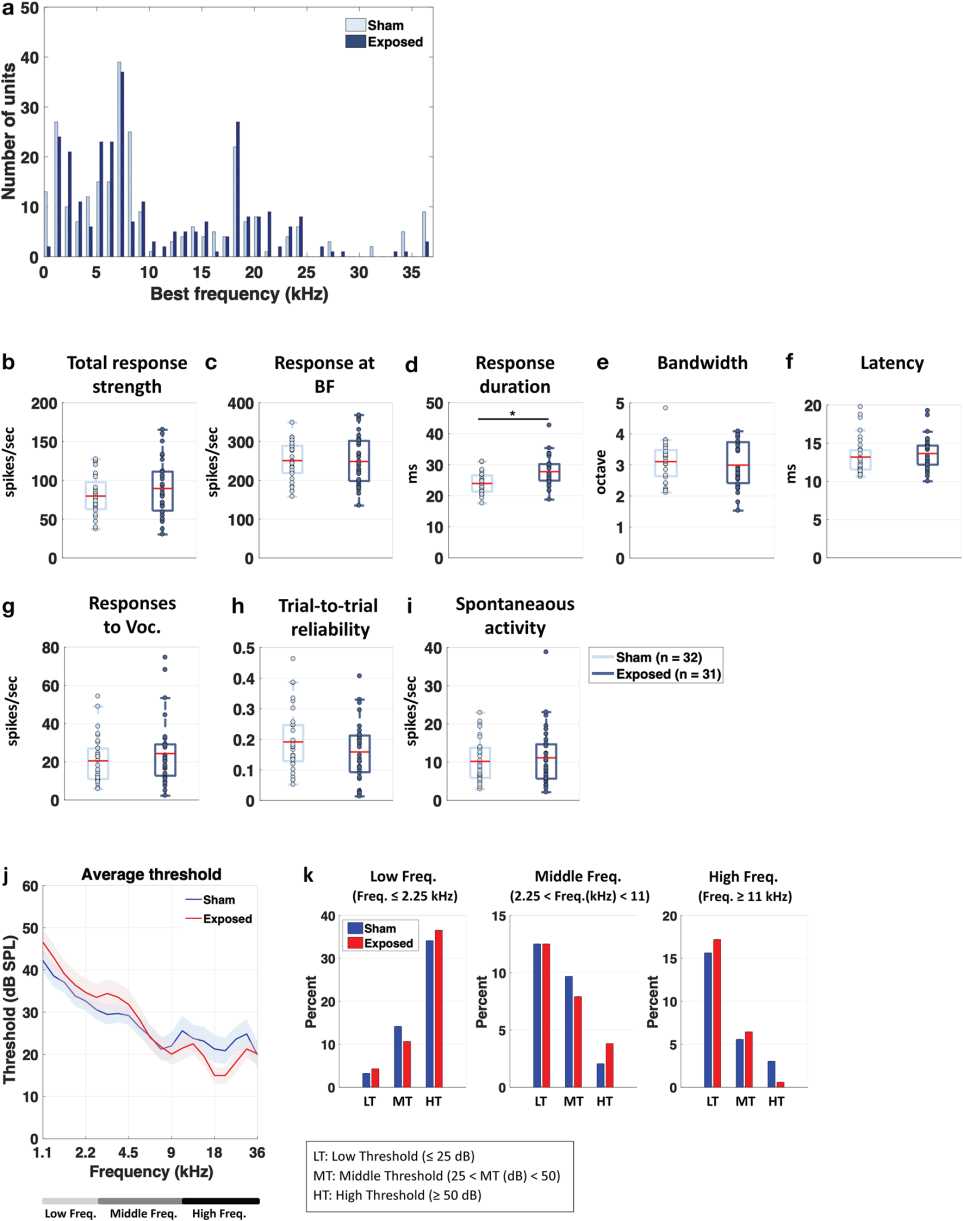
Effects of LTE exposure on parameters quantified from cortical responses in healthy animals. (**a**) BF distributions of the cortical neurons for healthy animals exposed to LTE (in dark blue) and Sham-exposed to LTE (in light blue). The two distributions did not differ. (**b**–**f**) Effects of LTE exposure on the parameters quantifying the spectro-temporal receptive fields (STRF). The response strength was not significantly changed both on the entire STRF and at the best frequency (**b**,**c**). The response duration was slightly increased (**d**) but, the response bandwidth and the bandwidth were unchanged (**e**,**f**). Both the strength and the temporal reliability of the responses to vocalizations were unchanged (**g**,**h**). Spontaneous activity was not significantly changed (**i**). (*p < 0.05 unpaired t-tests). (**j**,**k**) Effects of LTE exposure on the cortical thresholds. On average, the threshold were not significantly changed in LTE-exposed rats compared to Sham-exposed rats, but the exposed animals had slightly lower thresholds in the high frequencies.

Figure 5a presents the distributions of best frequencies (BF, eliciting the largest responses at 75 dB SPL) for the cortical neurons obtained in healthy animals for the Sham and Exposed group. Statistical analysis showed that these two distributions were similar (Chi-Square, p = 0.157) indicating that comparisons between the two groups can be performed without sampling bias.

Figure 5b–f show box plots representing the distributions and mean values (red lines) of the parameters derived from the STRFs for the two groups. In healthy animals, LTE exposure by itself induced little change in the mean values of the STRF parameters. Compared to the Sham group (light blue boxes vs. dark blue boxes for the exposed group), the LTE exposure changed neither the total response strength nor the response at the BF (Fig. 5b,c; unpaired t-tests, p = 0.2176, and p = 0.8696 respectively). There was also no effect on the spectral bandwidth and the latency (respectively p = 0.6764 and p = 0.7129) but there was a significant increase in response duration (p = 0.047). There was also no effect on the strength of responses to vocalizations (Fig. 5g, p = 0.4375), on the trial-to-trial reliability of these responses (Fig. 5h, p = 0.3412) and on the spontaneous activity (Fig. 5i; p = 0.3256).

Figure 5j shows the mean (± sem) thresholds for the healthy rats from 1.1 to 36 kHz. It did not reveal significant differences between the sham and exposed rats, except in the high frequencies (11–36 kHz) where the Exposed animals had slightly lower threshold (unpaired-t-test, p = 0.0083). This effect reflects the fact that, in the exposed animals, there was slightly more neurons with low and middle thresholds (and less with high thresholds) in this frequency range (Chi-square = 18.312, p = 0.001; Fig. 5k).

To summarize, when performed on the healthy animals LTE-exposure has no effect on the response strength to pure tones and complex sounds such as vocalizations. In addition, in healthy animals the cortical auditory thresholds were similar between Exposed and Sham animals, whereas in the LPS-treated animals, the LTE exposure led to large increases in cortical thresholds especially in low and middle frequency ranges.

## Discussion

Our study reveals that in adult male rats undergoing acute neuroinflammation, an exposure to LTE-1800 MHz with a local SAR_ACx_ of 0.5 W/kg (see “Methods”) resulted in significant decreases in the strength of sound-evoked responses recorded in the primary ACx. These changes in neuronal activity occurred without any clear alteration in the extent of the spatial domain covered by microglial cell processes. This effect of LTE on the strength of cortical evoked responses was not observed in healthy rats. The differences in neuronal responsiveness can be attributed to biological effects of LTE signal rather than sampling bias, given the similarity of best frequency distributions among recorded units in LTE-exposed and sham-exposed animals (Fig. 4a). In addition, the absence of change in response latency and bandwidth of spectral tuning in LTE exposed rats indicates that, most likely, the recordings were sampled from the same cortical layers, which were localized in the primary ACx rather than in secondary area.

To our knowledge, the effect of LTE signals on neuronal responses has not been reported previously. However, previous studies have documented the capacity of GSM-1800 MHz or 1800 MHz continuous wave (CW) to alter neuronal excitability, albeit with marked differences depending on the experimental approach. Recordings of snail ganglia shortly after an exposure to 1800 MHz CW at a SAR level of 8.2 W/Kg, showed reduction in the threshold for firing action potential and neuronal accommodation^31^. On the other hand, spike and bursting activities in primary neuronal cultures derived from rat brain were reduced by exposures to GSM-1800 MHz or 1800 MHz CW applied for 15 min at a SAR of 4.6 W/kg. This suppressive effect was only partly reversible over a 30 min period after the end of the exposure. Full silencing of neurons was reached at a SAR of 9.2 W/kg. Dose–response analyses showed that GSM-1800 MHz was more efficient than 1800 MHz CW in suppressing burst activity indicating that neuronal responses depends on RF signal modulation^32^.

In our setting, cortical evoked responses were collected in vivo 3 to 6 h after the end of a 2 h head-only exposure. In a previous study, we investigated the effect of GSM-1800 MHz at a SAR_ACx_ of 1.55 W/kg and found no significant effect on the sound-evoked cortical responses in healthy rats^28^. Here, the only significant effect induced in healthy rat by exposure to LTE-1800 at a SAR_ACx_ of 0.5 W/kg was a slight increase in response duration at pure tones presentation. This effect is difficult to explain as it was not accompanied by an increase in response strength indicating that this longer response duration occurred with the same total number of action potentials emitted by the cortical neurons. One explanation could be that the LTE exposure potentially reduced the activity of some inhibitory interneurons, as it is documented that in the primary ACx, feedforward inhibitions control the duration of pyramidal cell responses triggered by excitatory thalamic inputs^33–37^.

In contrast, in rats submitted to LPS-triggered neuroinflammation, there was no effect of LTE exposure on the duration of sound-evoked neuronal firing, but marked effects were detected on the strength of evoked responses. Indeed, compared to neuronal responses recorded in LPS sham-exposed rats, neurons of LPS-treated rats exposed to LTE displayed a decrease in response strength, an effect observed both at presentation of pure tones and natural vocalizations. The reduction in responses strength to pure tones occurred without narrowing the bandwidth of the spectral tuning at 75 dB, and as it occurred at all sound intensities, it induced an increase in acoustic threshold of cortical neurons in the low and middle frequencies.

The reduced strength of evoked responses indicates that the effects of LTE signal in LPS treated animals at a SAR_ACx_ of 0.5 W/kg is similar to that of the GSM-1800 MHz applied at a three time higher SAR_ACx_ (1.55 W/kg)^28^. As for GSM signals, it is possible that head exposure to LTE-1800 MHz reduces neuronal excitability of ACx neurons in rats undergoing LPS-triggered neuroinflammation. In line with this assumption, we also observed a decrease in trial-to trial reliability of neuronal responses to vocalizations (Fig. 4h) and a trend toward a reduction in spontaneous activity (Fig. 4i). However, it is difficult to determine in vivo if LTE signals reduce the neurons intrinsic excitability or reduce the synaptic inputs, which control neuronal responses in the ACx.

First, these weaker responses can result from an intrinsically reduced excitability of cortical cells following exposure to the LTE 1800 MHz. Supporting this view, GSM-1800 MHz and 1800 MHz-CW reduced bursting activities when applied directly to primary cultures of cortical rat neurons at SAR levels of 3.2 W/kg and 4.6 W/kg, respectively^32, 38^, but the threshold SAR levels required for significant reductions of the bursting activities were not specified. Arguing in favor of a decrease in intrinsic excitability, we also observed that spontaneous firing rate was lower in exposed than in sham-exposed animal.

Second, the LTE-exposure could also affect synaptic transmission either from thalamo-cortical synapses or from cortico-cortical synapses. It is now largely documented that in auditory cortex, the breadth of spectral tuning is not exclusively determined by afferent thalamic projections and that intracortical connections endow additional spectral inputs to a cortical site^39,40^. In our experiment, the fact that cortical STRFs display similar bandwidths in exposed and sham-exposed animals is an indirect indication that the impact of the LTE-exposure was not on the cortico-cortical connections. This also suggests that connections from other cortical areas exposed at SARs higher than those measured in the ACx (Fig. 2) are probably not responsible for the altered responses reported here.

Here, there was a larger proportion of cortical recordings showing high thresholds in LPS-exposed than in LPS-sham exposed animals. Given that it has been proposed that cortical acoustic thresholds are mainly controlled by the strength of thalamo-cortical synapses^39,40^, it can be suspected that thalamo-cortical transmission is partially reduced by the exposure, either at the pre-synaptic (decrease in glutamate release) or at the post-synaptic level (decrease in the number of receptors or in their affinity).

Similar to the effects of GSM-1800 MHz, LTE-induced alterations of neuronal responses occurred in the context of LPS-triggered neuroinflammation, the signature of this being a microglial reaction^22^. Current evidence indicate that microglia strongly impacts on the activity of neuronal networks both in the normal and in the pathological brain^41–43^. Their capacity to modulate neurotransmission relies not only on their production of compounds that potentialize, or limit, neurotransmission but also on the high motility of their cell processes. In the cerebral cortex, both enhancements and reductions in the activity of neuronal networks trigger rapid enlargement of microglia spatial domain due to the outgrowth of microglial cell processes^44,45^. In particular, microglia protrusions are recruited in the vicinity of activated thalamo-cortical synapses and can suppress the activity of excitatory synapses through a mechanism involving microglia-mediated local generation of adenosine^46^.

In LPS-treated rats submitted to GSM-1800 MHz at a SAR_ACx_ of 1.55 W/kg, the reduced activity of ACx neurons occurred together with an outgrowth of microglia processes marked by a significant increase in Iba1-stained area in the ACx^28^. This observation suggested that microglial reshaping triggered by GSM exposure could actively contribute to the GSM-induced reduction in sound-evoked neuronal responses. Our current study argues against this hypothesis in the context of a LTE-head exposure with a SAR_ACx_ limited to 0.5 W/kg, as we found no increase in the spatial domain covered by microglial processes. However, this does not rule out any effect of LTE signals on LPS activated-microglia, which could in turn affect neuronal activity. Further investigation is required to answer this question and to determine the mechanism by which acute neuroinflammation change the neuronal responsiveness to LTE signals.

To our knowledge the influence of LTE signals on auditory processing was not investigated previously. Our previous studies^26,28^ and the current one indicate that in the context of an acute inflammation, a head-only exposure to GSM-1800 MHz or LTE-1800 MHz can result in functional alterations of neuronal responses in ACx, as indicated by the increase in auditory threshold. For at least two main reasons, the cochlear function should be unaffected by our LTE exposure. First, as shown by the dosimetric study presented in Fig. 2, the highest levels of SAR (close to 1 W/kg) were in the dorso-medial cortex (below the antenna) and they decreased largely when moving toward the more lateral and ventral portions of the head. It can be estimated to be around 0.1 W/kg at the level of the rat pinna (and lower in the ear canal). Second, when the ears of guinea pigs were exposed for 2 months to GSM 900 MHz (5 days/week, 1 h/day, SAR between 1 and 4 W/kg), there was no detectable change in the amplitude of distorsion-product otoacoustic emissions and in thresholds of auditory brainstem responses^47^. Also, in healthy rats, repeated head exposure to GSM 900 or 1800 MHz at a local SAR of 2 W/kg did not affect the function of cochlear outer hair cells^48,49^. These results echo the data obtained in humans where investigations have shown that 10 to 30 min-exposures to EMF emitted by GSM mobile phones had no consistent effect on the auditory processing assessed at the cochlear^50–52^ or brainstem level^53,54^.

In our study, the LTE-triggered alterations in neuronal firing were observed in vivo, from 3 to 6 h after the end of the exposure. In a previous study focused on the dorso-medial part of the cortex, several effects induced by a GSM-1800 MHz observed 24 h after exposure were no longer detected 72 h after exposure. This was the case for the extension of microglial cell processes, the downregulation of Interleukin-1ß gene and the post-translational modification of AMPA receptors^26^. Considering the lower SAR value estimated here in the auditory cortex (0.5W/kg) compared with that in the dorso-medial area (2.94 W/kg^26^), it seems likely that the modifications in neuronal activity reported here were transient.

Our data should be put in perspective with respect to eligible SAR limits and estimates of actual SAR values reached in the cerebral cortex of mobile phone users. Current standards applied to protect the general public set the SAR limit to 2 W/kg for a local head or torso exposure to RF ranging between 100 kHz and 6 GHz RF^55^.

Dosimetric simulations have been performed using different human head models to determine RF power absorption in the head in general, or in the different tissues of the head during a mobile phone communication. Besides the diversity in the human head models, the simulations emphasize significant differences or uncertainties in the estimations of the energy absorbed in the brain according to anatomical or histological parameters such as the external or internal shape of the skull, the thickness or the water content of the different head tissues, which can strongly vary according to age, gender or across individuals^56–58^. Furthermore, cell phone features such as the internal location of the antenna and cell phone positions relative to the user’s head, strongly impact on the levels and distribution of SAR values in the cerebral cortex^59,60^. However, considering the reported SAR distribution over the human cerebral cortex, which were established with phone models emitting RF in the range of 1800 MHz^58–60^, it appears that SAR levels reached in the human auditory cortex remained less than half those applied in our study (SAR_ACx_ 0.5 W/kg). Therefore, our data do not challenge the current limitations in SAR values applied to the general public.

In conclusion, our study reveals that a single head-only exposure to LTE-1800 MHz can interfere with the neuronal responses of cortical neurons to sensory stimuli. In line with previous characterizations of the effect of GSM-signal, our results show that the impact of LTE signal on neuronal activity varies according to the health state. Acute neuroinflammation sensitize neuronal responses to LTE-1800 MHz, resulting in altered cortical processing of auditory stimuli.

## Methods

### Subjects

Data were collected in the cerebral cortex of 31 adult male Wistar rats obtained from Janvier Laboratories at 55 days of age. Rats were housed in a humidity (50–55%) and temperature (22–24 °C)-controlled facility on a 12 h/12 h light/dark cycle (light on at 7:30 A.M.) with free access to food and water. All experiments were conducted in accordance with the guidelines established by the European Communities Council Directive (2010/63/EU Council Directive Decree), which are similar to those described in the *Guidelines for the Use of Animals in Neuroscience Research of the Society of Neuroscience*. The protocol was approved by the ethical committee Paris-Sud and Centre (CEEA N°59, project 2014-25, national agreement 03729.02) using the procedures 32-2011 and 34-2012 validated by this committee.

The animals were habituated to the colony rooms for at least 1 week before LPS treatment and exposure (or sham-exposure) to LTE-EMF.

### LPS proinflammatory treatment

Twenty-two rats were injected intraperitoneally (i.p.) with *E. coli* LPS (250 µg/kg, serotype 0127:B8, SIGMA) diluted in sterile endotoxin-free isotonic saline 24 h before LTE or sham-exposure (n = 11 per group). In 2 month-old Wistar male rats, this LPS treatment generates a neuroinflammatory reaction, which in the cerebral cortex, is marked by upregulations of several proinflammatory genes (Tumor Necrosis Factor-alpha, Interleukin1ß, CCL2, NOX2, NOS2) 24 h after the LPS injection, including 4- and 12-fold increases in the levels of transcripts encoding the NOX2 enzyme and interleukin 1ß**,** respectively^26^. At this 24 h time point, cortical microglia display typical “bushy” cell morphologies expected from LPS-triggered proinflammatory activation of the cells (Fig. 1), which correspond to LPS-triggered proinflammatory activation of the cells documented by others^24,61^.

### Exposure to LTE-1800 MHz

Head-only exposure to LTE EMF was performed with an experimental setup previously used to assess the effect of GSM EMF^26^. LTE exposure was carried out 24 h after LPS injections (11 animals) or without LPS treatment (5 animals). The animals were lightly anaesthetized with Ketamine/Xylazine (Ketamine 80 mg/kg, i.p; Xylazine 10 mg/kg, i.p) before exposure to prevent movements and ensure reproducible position of the animals’ head below the loop antenna emitting the LTE signal. Half of the rats from the same cages served as control (11 sham-exposed animals, among the 22 rats pretreated with LPS): they were placed below the loop antenna with the energy of the LTE signal set to zero. The weights of the exposed and sham-exposed animals were similar (p = 0.558, unpaired t-test, ns). All the anesthetized animals were placed on a metal-free heating pad to maintain their body temperature around 37 °C throughout the experiment. As in previous experiments, the exposure duration was set at 2 h. After exposure, the animals were placed on another heating pad in the surgery room. The same exposure procedure was also applied to 10 healthy rats (untreated with LPS), half of them from the same cages were sham-exposed (p = 0.694).

### EMF exposure system

The exposure system was analogous to the one described in previous studies^25,62^, replacing the radiofrequency generator so as to generate LTE instead of GSM electromagnetic field. Briefly, a radiofrequency generator emitting a LTE − 1800 MHz electromagnetic field (SMBV100A, 3.2 GHz, Rohde & Schwarz, Germany) was connected to a power amplifier (ZHL-4W-422+, Mini-Circuits, USA), a circulator (D3 1719-N, Sodhy, France), a bidirectional coupler (CD D 1824-2, − 30 dB, Sodhy, France) and a four-ways power divider (DC D 0922-4N, Sodhy, France), allowing simultaneous exposure of four animals. A power meter (N1921A, Agilent, USA) connected to the bidirectional coupler allowed continuous measurements and monitoring of incident and reflected powers within the setup. Each output was connected to a loop antenna (Sama-Sistemi srl; Roma) enabling local exposure of the animal’s head. The loop antenna consisted of a printed circuit with two metallic lines engraved in a dielectric epoxy resin substrate (dielectric constant ε_r_ = 4.6). At one end, this device consisted of a 1 mm wide line forming a loop placed close to the animals’ head. As in previous studies^26,62^, specific absorption rates (SARs) were determined numerically using a numerical rat model with the Finite Difference Time Domain (FDTD) method^63–65^. They were also determined experimentally in homogeneous rat model using a Luxtron probe for measurement of rises in temperature. In this case, SARs, expressed in W/kg, were calculated using the following equation: SAR = C ΔT/Δt with C being the calorific capacity in J/(kg K), ΔT, the temperature change in °K and Δt, the time in seconds. Numerically determined SAR values were compared with experimental SAR values obtained using homogenous models, especially in the equivalent rat brain area. The difference between the numerical SAR determinations and the experimentally detected SAR values was less than 30%.

Figure 2a shows the SAR distribution in the rat brain with a rat model, which matched that of the rats used in our study in terms of weight and size. The brain averaged SAR was 0.37 ± 0.23 W/kg (mean ± std). SAR values were the highest in cortical areas located directly below the loop antenna. The local SAR in the ACx (SAR_ACx_) was 0.50 ± 0.08 W/kg (mean ± std) (Fig. 2b). As the weight of the exposed rats was homogenous, differences in tissue thickness at the level of the head were negligible, so the actual SAR in ACx or other cortical regions was expected to be very similar from one exposed animal to another.

### Surgical procedure for electrophysiological recordings

At the end of exposure, the animal was supplemented with additional doses of Ketamine (20 mg/kg, i.p.) and Xylazine (4 mg/kg, i.p.) until no reflex movement could be observed after pinching the hind paw. A local anesthetic (Xylocain 2%) was injected subcutaneously in the skin above the skull and on the temporal muscles, and the animal was placed on a metal-free heating system. After placing the animal in a stereotaxic frame, a craniotomy was performed above the left temporal cortex. The opening was 9 mm wide starting at the intersection point between parietal and temporal bones and 5 mm height as in our previous studies^66^. The dura above the ACx was carefully removed under binocular control without damaging the blood vessels. At the end of the surgery, a pedestal in dental acrylic cement was built to allow atraumatic fixation of the animal’s head during the recording session. The stereotaxic frame supporting the animal was placed in a sound-attenuating chamber (IAC, model AC1).

### Recording procedures

Data were from multiunit recordings in the primary auditory cortex of 20 rats including 10 animals pretreated with LPS. Extracellular recordings were from arrays of 16 tungsten electrodes (TDT, ø: 33 µm, < 1 MΩ) composed of two rows of 8 electrodes separated by 1000 µm (350 µm between electrodes of the same row). A silver wire (ø: 300 µm), used as ground, was inserted between the temporal bone and the dura mater on the contralateral side. The estimated location of the primary ACx was 4–7 mm posterior to Bregma, 3 mm ventral to the superior suture of the temporal bone. The raw signal was amplified 10,000 times (TDT Medusa) then processed by a multichannel data acquisition system (RX5, TDT). The signal collected from each electrode was filtered (610–10,000 Hz) to extract multi-unit activity (MUA). The trigger level was carefully set for each electrode (by a co-author blind to the exposed or sham-exposed status) to select the largest action potentials from the signal. On-line and off-line examination of the waveforms indicates that the MUA collected here was made of action potentials generated by 3 to 6 neurons at the vicinity of the electrode. At the beginning of each experiment, we set the position of the electrode array in such a way that the two rows of eight electrodes can sample neurons responding from low to high frequency when progressing in the rostro-caudal direction.

### Acoustic stimuli

Acoustic stimuli were generated in Matlab, transferred to a RP2.1-based sound delivery system (TDT) and sent to a Fostex speaker (FE87E). The speaker was placed at 2 cm from the rat’s right ear, a distance at which the speaker produced a flat spectrum (± 3 dB) between 140 Hz and 36 kHz. Calibration of the speaker was made using noise and pure tones recorded by a Bruel and Kjaer microphone 4133 coupled to a preamplifier B&K 2169 and a digital recorder Marantz PMD671. Spectro-temporal receptive fields (STRFs) were determined using 97 gamma-tone frequencies, covering eight (0.14–36 kHz) octaves presented at 75 dB SPL at 4.15 Hz in a random order. The frequency response area (FRA) was determined using the same set of tones and presented from 75 to 5 dB SPL in random order at 2 Hz. Each frequency was presented eight times at each intensity.

Responses to natural stimuli were also evaluated. In previous studies^28^, we observed that, regardless of the neurons’ best frequency (BF), rat vocalizations rarely elicit robust responses in ACx, whereas heterospecific ones (such as song birds or guinea pig vocalizations) often trigger robust and reliable responses across the entire tonotopic map. Therefore, we tested cortical responses to guinea pig’s vocalizations (whistle calls used in^36^ concatenated in a 1 s stimuli, presented 25 times).

Inserting an array of 16 electrodes in the cortical tissue systematically induces deformation of the cortex. We gave at least a 20-min recovery period to allow the cortex to return to its initial shape, then the array was slowly lowered. Spectro-Temporal Receptive Fields (STRFs) were used to assess the quality of our recordings and to adjust the electrodes depth. The recording depth was 300–700 µm, which corresponds to layer III/IV and the upper part of layer V^67^. When a clear tuning was obtained for at least 12 of the 16 electrodes, and that the stability of the recordings was satisfied for 15 min, the protocol started by presenting the acoustic stimuli in the following order: (1) Gamma-tones to determine the STRF at 75 dB SPL (5 min), (2) Gamma-tones at intensities ranging from 75 to 5 dB SPL (at 2 Hz) to determine the Frequency Responses Area (FRA, lasting 12 min), (3) vocalizations (25 repetitions) at 75 dB SPL (2 min). Presentation of the entire set of stimuli lasted 35 min, with periods of 2 min of spontaneous activity collected before and after these stimuli. This set of acoustic stimuli was used with the electrode array positioned at 3–5 locations per animal in the primary ACx.

### Data analysis

The STRFs derived from MUA were obtained by constructing post-stimulus time histograms (PSTHs) for each frequency with 1 ms time bins. All spikes falling in the averaging time window (starting at stimulus onset and lasting 100 ms) were counted. Thus, STRFs are matrices of 100 bins in abscissa (time) multiplied by 97 or 129 bins in ordinate (frequency). For visualization, all STRFs were smoothed with a uniform 5 × 5 bin window, but the actual values of response latency and bandwidth were quantified from raw data.

From the STRF obtained at 75 dB SPL, the Best Frequency (BF) was defined as the frequency (in kHz) where the highest firing rate was recorded. At each intensity, peaks of significant response were automatically identified using the following procedure: A positive peak in the MUA-based STRF was defined as a contour of firing rate above the average level of the baseline activity (estimated from the ten first milliseconds of STRFs at all intensity levels) plus six times the standard deviation of the baseline (spontaneous) activity. For a given cortical recording, four measures were extracted from the peak of the STRF.

First, the “response strength” was the total number of spikes falling in the significant peaks of the STRFs. Second, the “response duration” was the time difference between the first and last spike of the significant peaks. Third, the “bandwidth” was defined as the sum of all peaks width in octaves. Forth, the response latency was measured as the time at which the firing rate was 6 times above spontaneous activity (i.e. the latency of the significant contour).

The response to the natural stimulus (guinea pig whistle) was quantified (i) by the firing rate (number of action potentials) emitted during the 345 ms of presentation of that stimulus and (ii) the trial-to-trial temporal reliability coefficient (CorrCoef) which quantifies the trial-to-trial reliability of the response over the 20 repetitions of the same stimulus. This index corresponds to the normalized covariance between each pair of spike trains recorded at presentation of this vocalization and was calculated as follows:

$$CorrCoef = \frac{1}{{{\text{N}}\left( {{\text{N}} - 1} \right)}}\sum\limits_{i = 1}^{N - 1} {\sum\limits_{j = i + 1}^{N} {\frac{{\sigma_{{x_{i} x_{j} }} }}{{\sigma_{{x_{i} }} \sigma_{{x_{j} }} }}} }$$

where N is the number of trials and *σ*_*xixj*_ is the normalized covariance at zero lag between spike trains x_i_ and x_j_ where i and j are the trial numbers. Spike trains x_i_ and x_j_ were previously convolved with a 10-ms width Gaussian window, as in previous studies^36,68–72^.

### Immunohistochemistry

Eleven LPS-treated rats, submitted to identical LTE or sham exposure protocols (n = 6 or 5 per group), were used for immunohistochemistry in the cerebral cortex. Three to four hours after exposure (or sham exposure), adult rats were deeply anaesthetized with pentobarbital and perfused with 0.1 M phosphate buffered saline (PBS) followed by paraformaldehyde 4% in 0.1 M PBS, pH 7.4. Brains were post-fixated overnight in paraformaldehyde 4%, then cryoprotected by immersion in PBS containing 25% (w/v) sucrose and frozen in − 70 °C isopentane. Immunostaining was performed on coronal sections (20 µm thick) cut on a cryostat (Microm, Heidelberg, Germany) and mounted on gelatin-coated Superfrost glass slides (Menzel-glazer, Freiburg, Germany). Sections were blocked in PBS containing 0,1% Triton-X100 and 10% goat serum (30 min at room temperature) and then incubated overnight at 4 °C with rabbit polyclonal anti-Iba1 (1:400, Wako Chemicals). Bound antibodies were detected by applying biotinylated goat anti-rabbit IgG (GE Healthcare, Velizy Villacoublay, France) for 2 h at room temperature, followed by Alexa Fluor 488-conjugated streptavidin (Life technology, Saint Aubin, France) for 30 min at room temperature. Sections were counterstained with Hoechst 33342 dye (2 µg/ml) and mounted in fluoromount G (Clinisciences). Control staining, performed by omitting primary antibodies, was negative.

### Image analysis

Quantitative image analyses were performed as described previously^73^. Images were captured using a Zeiss upright widefield microscope equipped with the apotome system. A 10× NA 0.50 Fluar objective (Zeiss microscope) was used to acquire images with equal exposure times. For each animal and each cortical region assessed, immunostained microglial cells were analyzed in microscopic fields (6 × 10^5^ µm^2^ area) acquired in 12 sections spanning areas of the auditory cortex and distributed over a cortical region extending in the rostro-caudal axis between 3.5 and 6.4 mm caudal to the bregma. Image analysis was performed blind relative to the experimental groups. The microglial cell bodies, confirmed by nuclei counterstaining, with Hoechst dye were counted manually. The area stained by anti-Iba1 and that of Iba1-stained cell bodies were determined following assignment of a threshold to eliminate background immunofluorescence, using Image J software.

### Statistical analysis

Differences between groups of animals were analyzed using non-parametric Chi-square test or parametric unpaired Student t-test. Unpaired t-test was applied checking the assumption of equal variances and Gaussian distributions with F-test and Kolmogorov and Smirnov (K-S) test using Prism software.

### Ethical approval

All procedures and experiments were conducted in accordance with the guidelines established by the European Communities Council Directive (2010/63/EU Council Directive Decree), which are similar to those described in the *Guidelines for the Use of Animals in Neuroscience Research of the Society of Neuroscience*. The protocols were approved by the institutional ethic committee Paris-Sud and Centre (CEEA N°59, project 2014-25, national agreement 03729.02) using the procedures 32-2011 and 34-2012 validated by this committee. We further attest that all efforts were made to minimize the number of animals used and their suffering. This study was performed in accordance with ARRIVE guidelines.

## Data Availability

Data used in this study are available on reasonable request.
